# Site-occupancy factors in the Debye scattering equation. A theoretical discussion on significance and correctness

**DOI:** 10.1107/S2053273323008446

**Published:** 2023-11-02

**Authors:** Fabio Ferri, Maria Chiara Bossuto, Pietro Anzini, Antonio Cervellino, Antonietta Guagliardi, Federica Bertolotti, Norberto Masciocchi

**Affiliations:** a Dipartimento di Scienza e Alta Tecnologia & To.Sca.Lab, Università degli Studi dell’Insubria, via Valleggio 11, Como, 22100, Italy; b Swiss Light Source, Paul Scherrer Institute, Villigen PSI, 5232, Switzerland; c Istituto di Cristallografia (IC) & To.Sca.Lab, Consiglio Nazionale delle Ricerche (CNR), via Valleggio 11, Como, 22100, Italy; Czech Academy of Sciences, Czech Republic

**Keywords:** Debye scattering equation, site-occupancy factors, defective nanocrystals

## Abstract

The Debye scattering equation (DSE) [Debye (1915). *Ann. Phys.* **351**, 809–823] is widely used for analyzing total scattering data of nanocrystalline materials in reciprocal space. In its modified form (MDSE) [Cervellino *et al.* (2010). *J. Appl. Cryst.* **43**, 1543–1547], it includes contributions from uncorrelated thermal agitation terms and, for defective crystalline nanoparticles (NPs), average site-occupancy factors (s.o.f.’s). The s.o.f.’s were introduced heuristically and no theoretical demonstration was provided. This paper presents in detail such a demonstration, corrects a glitch present in the original MDSE, and discusses the s.o.f.’s physical significance. Three new MDSE expressions are given that refer to distinct defective NP ensembles characterized by: (i) vacant sites with uncorrelated constant site-occupancy probability; (ii) vacant sites with a fixed number of randomly distributed atoms; (iii) self-excluding (disordered) positional sites. For all these cases, beneficial aspects and shortcomings of introducing s.o.f.’s as free refinable parameters are demonstrated. The theoretical analysis is supported by numerical simulations performed by comparing the corrected MDSE profiles and the ones based on atomistic modeling of a large number of NPs, satisfying the structural conditions described in (i)–(iii).

## Abstract 2

The modified Debye scattering equation, often used for characterizing defective nanoparticle ensembles, has been theoretically analyzed with a special focus on the significance and correctness of the site-occupancy factors, *o*_*i*_’s.

## 1. Introduction

Powder diffraction (PD) is a powerful tool that has been used for decades in many fields of science and technology (chemistry, materials science, metallurgy, geology, forensic science, cultural heritage) and is nowadays commonplace in both academic and industrial sectors. Thanks to the availability of high-quality data and the development of advanced computational tools, PD is widely used for studying not only polycrystalline materials with large crystalline domains and sharp Bragg peaks, but also defective and/or nano-sized materials where both (broadened) Bragg peaks and diffuse scattering present in between the peaks carry relevant information about the material structure and defectiveness, and size and shape of nanocrystals. The analysis of the total (peaks + diffuse) elastic scattering data is customarily carried out in the reciprocal space by using the Debye scattering equation (DSE) or in the real space by recovering the pair distribution function (PDF) via a Fourier transform of the scattering data. Recent reviews on the two complementary techniques have been carried out by Scardi & Gelisio (2016 ▸), Billinge (2019 ▸) and Cervellino *et al.* (2016 ▸).

During the last decade, the DSE approach was further refined by introducing the effects of atomic thermal motion and partial site-occupancy factors (s.o.f.’s). As a result, a modified version of the DSE (MDSE) was proposed and implemented in the open-source suite of programs known as *DEBUSSY* (Cervellino *et al.*, 2010 ▸, 2015 ▸). The introduction of s.o.f.’s in the MDSE allowed us to compute (exactly) the average scattered intensity *I*(*Q*) of an ensemble of defective nanoparticles (NPs) with randomly distributed vacancies in a very efficient way. This task would have been much more time consuming (and occasionally less accurate) if the computation had been carried out by averaging the *I*(*Q*)’s of many defective NPs with the vacant sites omitted. Thus, the MDSE allows the usage of s.o.f.’s as free adjustable parameters with an efficient fitting procedure, which is fast and highly accurate from a statistical point of view. The MDSE has been intensively adopted for the characterization of many nano-sized and defective species, including metals, oxides, ionic and organometallic species, up to colloids (Bertolotti *et al.*, 2018 ▸).

However, in spite of its wide and successful use, the MDSE was never validated from a theoretical point of view regarding the atomic s.o.f. parameters, which were introduced heuristically, and no theoretical demonstration was ever provided. In this paper we detail such a demonstration, correct a glitch present in the original MDSE, and discuss the s.o.f. physical significance, providing three new MDSE expressions that refer to NP defectiveness characterized by vacant sites with: (i) uncorrelated constant site-occupancy probability, or (ii) a fixed number of randomly distributed atoms, or (iii) self-excluding disordered positional sites (split-atom model). For all these cases, we provide both theoretical and numerical evidence of the effectiveness of introducing s.o.f.’s as free refinable parameters by comparing the new MDSE profiles and the ones based on atomistic modeling of a large number of NPs, satisfying the structural conditions described in (i)–(iii). Based on the theoretical approach here presented, we further correct the original MDSE, showing that the s.o.f.’s appearing in the self-term summation [*I*_self_(*Q*), see below] of the corrected MDSE should not be squared. This minor change only affects the smooth continuous behavior of *I*_self_(*Q*) and does not modify the *I*_dist_(*Q*) term of the original MDSE, where the structural NP information is encoded.

## 2. Theoretical background: the DSE

The DSE proposed by Debye (1915 ▸) describes the elastic scattering intensity distribution of randomly oriented monodisperse (*i.e.* of equal size, morphology and structure) non-interacting NPs, each composed of *n* atoms whose fixed positions in an arbitrary reference frame are known (regardless of any periodicity and order). The DSE reads

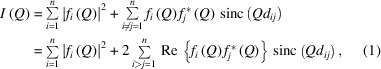

where sinc(*x*) = 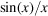 , 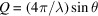 is the magnitude of the scattering vector, θ is half of the scattering (*i.e.* deflection) angle, λ is the radiation wavelength, 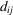 is the Euclidean distance between atoms *i* and *j*, and 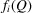 is the X-ray atomic form factor of the *i*th atom. Note that the atomic form factors are complex functions whereas, as shown by the second line of equation (1), the overall intensity is a real quantity. In the case of X-ray radiation, 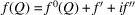 , where 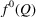 is the (real) elastic scattering term, and 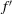 and 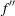 are the real and imaginary parts, respectively, of the anomalous scattering contribution due to atomic electron binding, which is *Q* independent but varies with radiation energy *E*. The behaviors of elemental 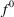 , 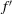 and 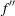 as a function of *Q* and *E* can be found in the work of Cullen *et al.* (1989 ▸). As a historical remark, we point out that in the original DSE form, the form factors were considered real because at that time (1915) X-ray anomalous scattering effects were not known [first shown by Mark & Szilard (1925 ▸)].

The first term of equation (1), which corresponds to the *self-scattered* intensity 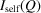 ( 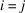 ), is given by the sum of the intensities scattered from all the atoms making up the NP; the second term, which is the *distinct-scattered* intensity 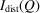 ( 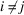 ), takes into account the interference between all pairs of distinct atoms within the NP. Therefore, 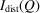 depends on the relative arrangement of the atoms and provides information on the structural features of the NP.

## 3. The modified Debye scattering equation

There are two main limitations of equation (1), which have prompted the development of a modified Debye scattering equation (MDSE).

The first one is the assumption of fixed atomic positions, thus neglecting the effects of unavoidable thermal vibrations. As done in conventional crystallography, the latter are usually taken into account by multiplying the form factors present in the distinct term of equation (1) by the Debye–Waller (DW) thermal factors (Warren, 1990 ▸) associated, in a simplified picture, to each atomic species *s* present in the NP. The atomic sites are then understood as the spatial averages of the vibrating atomic positions (equilibrium positions). In the case of isotropic and independent atomic vibrations, the DW factors associated to the *s*th species are given by 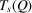 = 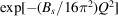 , where the thermal factor 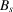 is related to the mean-square displacement 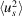 of all the atoms belonging to the *s* species about their equilibrium position by the relation 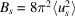 .

The second limitation of equation (1) is the difficulty of properly dealing with defective NP ensembles characterized by crystalline order but partial s.o.f.’s, where not all the sites that are present in the corresponding non-defective NPs are occupied by atoms. Thus, in spite of the assumption of identical NPs, the set of actual distances in equation (1) are not the same for all the NPs of the ensemble because, for each defective NP, the atoms are randomly distributed among the available sites. The observed profile 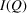 of such a system corresponds to the ensemble average of the intensities 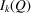 scattered by (many) defective, randomly oriented NPs. If we indicate with 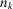 the actual number of atoms inside the *k*th NP of the ensemble, we can use equation (1) and write

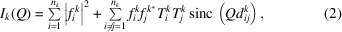

where we have introduced the DW thermal factors and, to help readability, we have omitted the *Q* dependence of the form factors and thermal factors.

Let us assume that all the NPs of the ensemble are characterized by the same available sites labeled with the indexes *i* and *j* 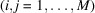 , and that each site *i* is assigned to a single atomic species through a function 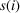 . If we define a binary variable 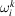 , that assumes the values 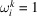 for occupied sites and 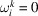 for vacant sites, we can re-write equation (2) as

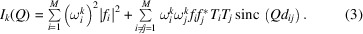

Note that, though 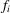 and 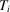 depend only on the species, to keep the notation simple, we have indicated only their (implicit) dependence on *i* and *j*, meaning that 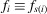 and 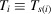 .

Since 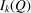 describes the intensity profile of the defective *k*-NP, the variables 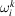 are intended as a single realization of the stochastic variables 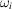 that assume the values 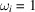 with probability 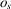 [which depends only on the species 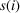 ] and 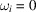 with probability 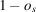 . Note that for non-defective NPs (all sites occupied) the variables 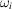 become deterministic with constant values 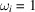 and correspondingly 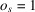 . The average of 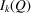 over the entire defective NP ensemble reads

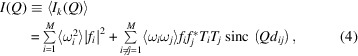

where 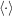 indicates the ensemble average. Equation (4) shows that 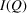 depends on the second moment 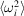 and on the second-order correlation 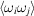 , which, in turn, are related to the type of defectiveness associated to the NP ensemble. In the following, starting from equation (4), we will work out the analytical expression for 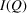 for three specific cases of NP defectiveness.

### 3.1. Defective NPs with uncorrelated constant occupancy probabilities

Let us consider a defective NP ensemble where all the s.o.f.’s of a given atomic species *s* are characterized by the same *constant occupancy probability* 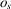 . Thus, the probability that any site *i* assigned to the species 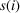 is occupied is equal to 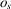 , whereas 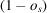 is the probability that the site is vacant. Let us further suppose there is no spatial correlation between the occupancies of the different sites. As a consequence of these assumptions, the number of atoms 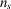 belonging to the *s* species present among the 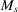 sites available for that species is not constant, but is a stochastic variable described by a binomial distribution

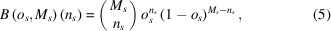

where 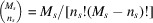 is the binomial coefficient. For such a binomial distribution, the average number of atoms is 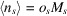 and the variance is 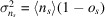 .

Under these hypotheses and by using the property that, for a binary variable 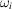 any moment 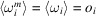 ( 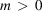 ), the average quantities inside equation (4) read

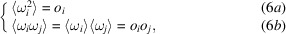

where 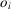 represents the occupancy probability of the *i*th site, which depends only on the atomic species 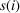 , and we have adopted the simplifying notation 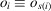 .

Upon substitution of equations (6) into equation (4), we get

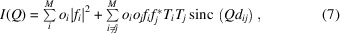

where 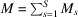 , *S* being the number of different species. Equation (7) represents a modified version of the original DSE (MDSE), in which both the DW thermal factors and the s.o.f.’s have been included. Clearly, when all the species are characterized by unitary occupancies 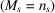 , the total number of available sites is equal to the total number of atoms (*M* = *n*) and aside from the DW factors, equation (7) reduces to equation (1).

It should be pointed out that a different MDSE version was proposed (and implemented in the software package *DEBUSSY*) by Cervellino *et al.* (2010 ▸). That version of the MDSE contains a glitch in the 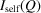 term, where the s.o.f.’s are (erroneously) squared. However, this error only affects the continuous, monotonic behavior of 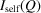 , without modifying the 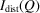 term, where all the structural features are encoded. Therefore, the presence of this glitch introduces only negligible effects on the NP structural characterization and, as will be shown in Section 5.1, it does not significantly affect any of the previously published results, where the total scattering data were interpreted according to the incorrect MDSE equation. A quantitative comparison between the original MDSE and our corrected version [equation (7)] is reported in Sections 4.1 and 4.2.

### 3.2. Defective NPs with a constant number of atoms

Let us consider a defective NP ensemble where each NP is characterized, species by species, by a *constant number* 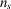 of atoms randomly distributed among the 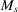 available sites ( 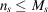 ). Thus, for all the sites *i* assigned to the species *s*, 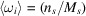 , but differently from Section 3.1, the assumption of independent occupancies remains valid only for sites belonging to different species. Since the average 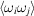 is equal to the probability of having 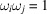 (which occurs only when 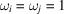 ), for sites of the same species we have

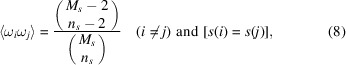

where the binomial coefficient in the denominator represents the number of combinations that can be realized by placing 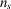 atoms within 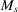 sites, and the one in the numerator is the number of combinations left available after the *i*th and *j*th sites have been occupied by two atoms, which occurs only when 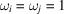 . By developing the two binomial coefficients of equation (8), and adopting the same simplified notation used in equations (6) [namely 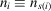 and 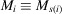 ], we end up with the result

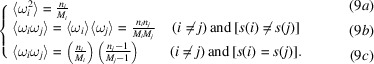




Provided that 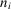 is equal to the average number of atoms of a defective NP ensemble with constant occupancy probability ( 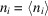 ), equations (9) are quite similar to equations (6). The only slight difference is in the right-hand term of equation (9*c*) which is (marginally) smaller than in equation (6*b*), implying that the constraint of constant number of atoms introduces a slightly negative (constant) correlation among the occupancies of the same atomic species. Indeed, since 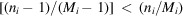 , the covariance 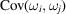 = 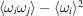 associated to equation (9*c*) is slightly negative. By inserting equations (9) into equation (4), we get

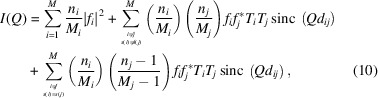

where 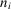 and 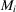 represent the number of atoms and sites of the 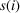 species, respectively, and *M* is the total number of sites. Under realistic conditions, *i.e.* for nano-sized NPs with 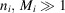 , equation (10) is almost identical to equation (7) because 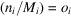 and 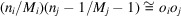 . We anticipate here that the difference between the two equations (and therefore between the two types of defectiveness) shows up only in the small-angle X-ray scattering (SAXS) region where the influence of the number of fluctuations present in equation (7) introduces an extra contribution, which is absent in equation (10). A quantitative comparison between these two equations is deferred to Section 5.2.

### 3.3. Defective NPs with self-excluding positional sites (split-atom model)

In this example we report a special case of correlated occupancies, where the constituent sites belonging to a given atomic species *s* can be split into different subsets (or clusters), each of them characterized by a given number 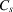 of (usually symmetry-related) crystallographic site positions. Since all the distances between the split site positions of a given cluster are typically smaller than a physically meaningful minimal distance (for example, the atom size), only one atom can be randomly placed within the 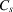 self-excluding crystallographic sites of that species (‘split-atom’ model).

**Figure 1:**
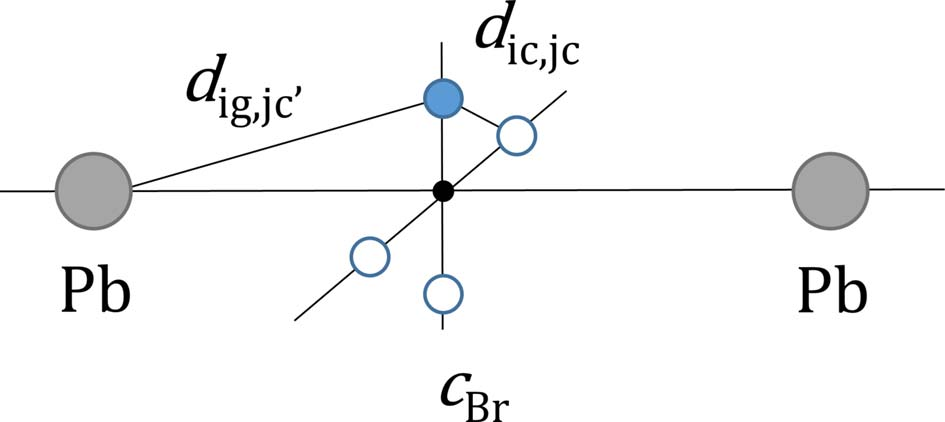
Schematic detail of a locally disordered *A*PbBr_3_ perovskite cluster where the Br atom, instead of occupying the highly symmetric position midway between the two Pb atoms (small black circle), is randomly located at one of the four split locations belonging to the *c*_Br_ cluster, that lie on the plane normal to the Pb⋯Pb vector.

As an example of where a subset of sites can host one atom only (of a single species), we discuss here the so-called ‘split-cubic’ model (Mashiyama *et al.*, 1998 ▸) that was proposed for disordered lead halide perovskites (*A*Pb*X*_3_, *A* = a monovalent cation, *X* = Cl, Br, I). Here, considering the case *A* = Cs, *X* = Br, the Br atom joining two Pb atoms occupies one of the four different positions that, with respect to its ideal location (midway along the Pb⋯Pb vector, taken as the [100] edge of the unit cell aligned with *x*, with a Pb—Br—Pb angle of 180°), are shifted up–down–left–right in the (002) plane by some 0.3–0.4 Å in the ±*y* or ±*z* directions. This situation, clearly visible in Fig. 2 of Mashiyama *et al.* (1998 ▸), is sketched in Fig. 1 ▸ where three of the four Br sites are (*must be*) vacant (white circles), whereas only one site (blue circle) contains (*must contain*) a Br atom. Other examples of self-excluding positional disorder can be found in the literature, either with smaller [ 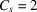 , as in hexagonal hy­droxy­apatite (Leeuw, 2001 ▸)] or larger [ 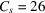 , as in another Pb-containing perovskite (Cervellino *et al.*, 2011 ▸)] multiplicities.

In our example, 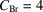 , whereas 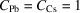 , meaning that the Pb and Cs sites are not split. Thus, the site occupancies are uncorrelated only when different clusters are considered. Conversely, the occupancies are fully (anti)correlated for atoms within the same cluster (in the example of Fig. 1 ▸, the Br cluster, 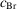 ), because only one site of the cluster is occupied.

As in the previous cases, let us indicate with 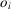 the occupancy probability of the *i*th site assigned to the 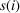 species. Thus, if we assume that all the NPs of the ensemble have a constant number of atoms and there is no spatial correlation between the occupancies of sites belonging to different clusters, we can immediately write

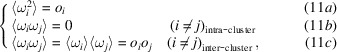

where 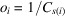 . Note that the number of atoms of the unsplit species is equal to the number of corresponding sites ( 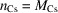 and 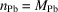 ), whereas for the split species 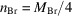 , implying that 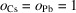 and 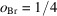 . Although equations (11) refer to NPs with a constant number of atoms (as in Section 3.2) and unitary occupancies for unsplit species, its generalization to partial uncorrelated occupancies is rather straightforward.

Upon substitution of equations (11) into equation (4), we obtain

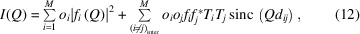

where the double sum of the distinct term is restricted only to the inter-cluster distances. Note that, except for the restricted sum of the distinct term, equation (12) is formally identical to equation (7), thus extending the application of the (correct) MDSE from defective nanocrystals with vacant sites *only*, to those with self-excluding disordered positional sites.

Another way of recasting equation (12) is by using the Heaviside Θ function, defined as 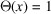 for 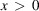 , 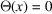 for 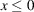 . This version is computationally efficient and is the one actually implemented (apart from the correction to the self-scattering term) in the *DEBUSSY* software suite,

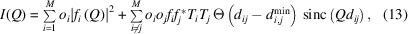

where 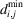 is the minimal distance allowed between atoms of species 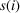 and 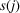 and, as above, we have adopted the simplifying notation 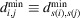 . DSE computation through equation (13) is faster than using equation (12) because it does not need a pre-classification of atoms into clusters, just a look-up table for the possible minimal bond distances for each atomic species pair. Then all pairs with distances shorter than 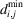 are simply ignored.

## 4. Numerical simulations

To crosscheck the validity of the analysis outlined in the previous section, we carried out several numerical simulations corresponding to single- and multi-species defective NP ensembles selected as case studies. In the following, we report three examples of such numerical simulations. Owing to computational issues intrinsic to the DSE calculation from scratch, our analysis has been limited to ultra-small NPs, without any lack of generality.

### 4.1. Case 1: elemental f.c.c. Au nanocrystals with constant vacant site probability

For the first example, we selected an ideal monoatomic nanocrystal (NC), namely a f.c.c. (face-centered cubic) Au NC (*a* = 4.080 Å), in the form of a cube with six unit cells per edge, characterized by *M* = 1099 sites, side *L* = 2.45 nm and equivalent diameter (of the sphere of equal volume) 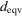 = 3.04 nm. Then, we generated many defective NCs by filling the *M* sites with 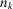 atoms that were randomly spread among the sites. The numbers 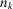 were extracted stochastically from a binomial distribution 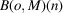 with a given occupancy probability *o*. To emphasize the differences between the original and corrected MDSE, we intentionally selected a fairly small (and unrealistic) occupancy probability, *i.e.* 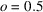 . Then, for each defective NC, we computed 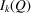 by using equation (2) (with *B* = 0.5 Å^2^ or 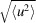 = 7.96 × 10^−2^ Å) and averaged such a profile over a large number 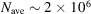 of NCs until the average profile 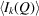 was determined with very high accuracy 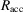 = 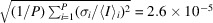 , where the sum runs over all the 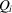 points ( 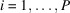 ) of the intensity profile and 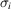 is the standard deviation associated to 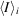 .

**Figure 2:**
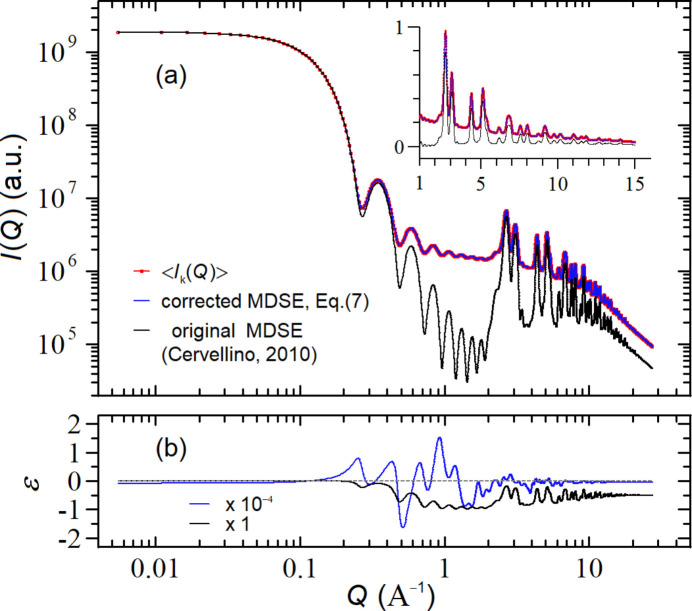
(*a*) Comparison between the *I*(*Q*) profiles computed with the corrected MDSE [equation (7), blue curve] and the original MDSE [(Cervellino *et al.*, 2010 ▸), black curve] against the expected 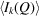 obtained by averaging the intensity profiles of many defective NCs in the case of a f.c.c. Au NC (*a* = 4.08 Å) made of 6 × 6 × 6 unit cells (side *L* = 2.45 nm) with an average occupancy factor 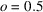 ; in the inset the same (rescaled) data are shown on a (reduced range) linear plot. (*b*) Relative deviations ɛ between the corrected and the original MDSE against the expected 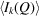 profiles, ɛ = [*I*(*Q*) − 〈*I_k_* (*Q*)〉]/〈*I_k_* (*Q*)〉. The factors appearing in the legend indicate the absolute scales of the *y*-axis values.

Fig. 2 ▸(*a*) compares 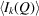 (red symbols) with the profiles 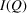 computed by using the original MDSE [(Cervellino *et al.*, 2010 ▸), black curve] and the corrected MDSE [equation (7), blue curve]. The same (rescaled) profiles are shown in the inset of Fig. 2 ▸(*a*) on a linear plot. As is evident, the original MDSE is not capable of reproducing 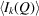 over most of the *Q* range. Conversely, the match between the 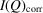 profile calculated by using the corrected MDSE and 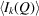 is almost perfect [not distinguishable in Fig. 2 ▸(*a*)], proving the correctness of equation (7). The small relative deviations ɛ between these two curves {ɛ = 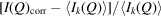 }, shown in Fig. 2 ▸(*b*), can be altogether quantified by the (dis)agreement parameter

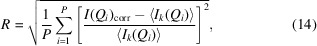

which, in the case of the blue and red curves of Fig. 2 ▸(*a*), is 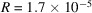 , a figure that is consistent with the accuracy associated to 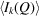 .

Interestingly, Fig. 2 ▸(*a*) shows that at large *Q*’s there is a factor ∼2 between the corrected and original MDSE. This occurs because, for 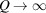 , the main contribution to 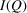 comes from 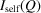 and the ratio 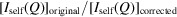 = *o* = 0.5. Conversely, at small *Q*’s, the original and corrected MDSE are almost superimposed, consistent with the fact that, for 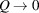 , the main contribution to 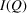 comes from the 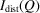 term, which is the same in the original and corrected MDSEs. As a final comment, we point out that the Bragg peaks of the original MDSE appear to be much more pronounced than those of the corrected one due to the (erroneously) reduced 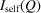 contribution present in the original MDSE.

As anticipated, the presented f.c.c. Au phase with 50% of randomly positioned vacancies is clearly a non-physical system, prone to collapse into a (twice as) denser phase. However, the same data analysis approach used here can be employed to analyze randomly mixed alloys when the scattering contrast of the constituent elements is rather high. This is valid for example for any f.c.c. Ni_*x*_V_1−*x*_ alloy [*x* = 0.75 (Koester & Gmoehling, 1960 ▸); *x* = 0.60 (Pearson & Hume-Rothery, 1952 ▸)], if studied by neutron radiation. In this case, the coherent scattering lengths for natural abundance Ni and V nuclei are 10.4 and −0.38 fm, respectively (Sears, 1992 ▸). More practically, random nano-alloys of Au_*x*_Ag_1−*x*_, largely studied for their catalytic and surface plasmonic states (Newmai *et al.*, 2022 ▸; Kozioł *et al.*, 2021 ▸; Coviello *et al.*, 2022 ▸), can also be studied by similar data analysis protocols, but this analysis is beyond the purpose of this article.

### 4.2. Case 2: non-stoichiometric cubic PbS nanocrystals with constant vacant site probability

**Figure 3:**
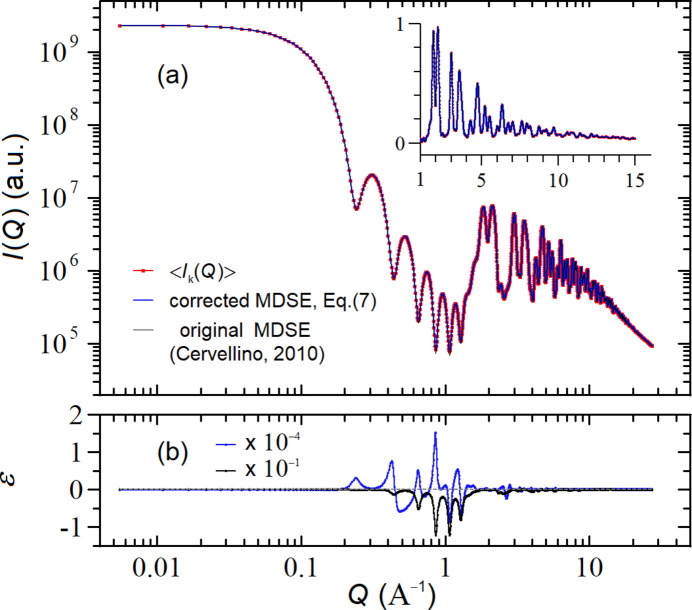
(*a*) Comparison between the *I*(*Q*) profiles computed with the corrected MDSE [equation (7), blue curve] and the original MDSE [(Cervellino *et al.*, 2010 ▸), black curve] against the expected 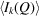 obtained by averaging the intensity profiles of many defective NCs in the case of a PbS NC (*a* = 5.924 Å) made of 5 × 5 × 5 unit cells (*L* = 2.67 nm) with average occupancies 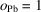 and 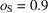 ; in the inset the same (rescaled) data are shown on a (reduced range) linear plot. (*b*) Relative deviations 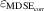 and 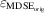 . The factors appearing in the legend indicate the absolute scales of the *y*-axis values.

In the second example, we selected a biatomic NC, namely a PbS NC (*a* = 5.924 Å) made of 5 × 5 × 5 unit cells, which corresponds to a cubic crystal characterized by *M* = 1000 sites ( 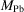 = 500, 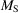 = 500), side *L* = 2.67 nm and equivalent diameter 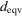 = 3.67 nm. Then, we followed the same procedure described for the Au NC, but in this case we selected less extreme (Bertolotti *et al.*, 2016 ▸) occupancies, 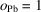 and 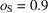 , and obtained 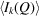 with 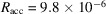 ( 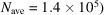 . Analogously to what was done in the previous subsection, Fig. 3 ▸(*a*) compares the simulated 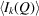 (red symbols) with the 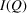 computed by using the original MDSE [(Cervellino *et al.*, 2010 ▸), black curve] and with the corrected MDSE [equation (7), blue curve]. As one can easily appreciate, the three curves are almost perfectly superimposed, both on the main plot (log–log scale) and on the inset (linear scale), showing that, under these working conditions, the effects of the corrected MDSE are rather marginal. Nevertheless, the difference is evident in the residual plots of Fig. 3 ▸(*b*) near *Q* = 1 Å^−1^, where 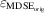 and 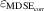 differ by more than three orders of magnitude. Correspondingly 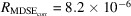 , whereas 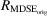 = 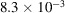 .

### 4.3. Case 3: stoichiometric CsPbBr_3_ perovskite with self-excluding positional sites (split-cubic model)

In the last example we considered a cubic CsPbBr_3_ perovskite NC (*a* = 5.927 Å), made of 5 × 5 × 5 unit cells with a resulting side *L* = 2.67 nm where the sites corresponding to the Cs and Pb atoms are completely occupied ( 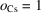 , 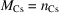 = 125 and 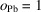 , 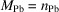 = 125), whereas the Br atoms are randomly displaced crosswise by 0.5 Å normally from the center of the Pb⋯Pb vector (see Fig. 1 ▸), maintaining an average holoedric cubic symmetry with a distance 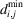 = 0.71 Å. Thus, the number of Br atoms is 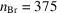 and the corresponding self-excluding positional sites are 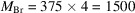 , with occupancy 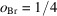 . For each defective NC, we computed 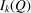 (for simplicity, with all atoms at rest, *i.e.* 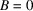 ) and averaged such a profile until 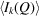 was determined with 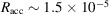 ( 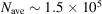 ).

**Figure 4:**
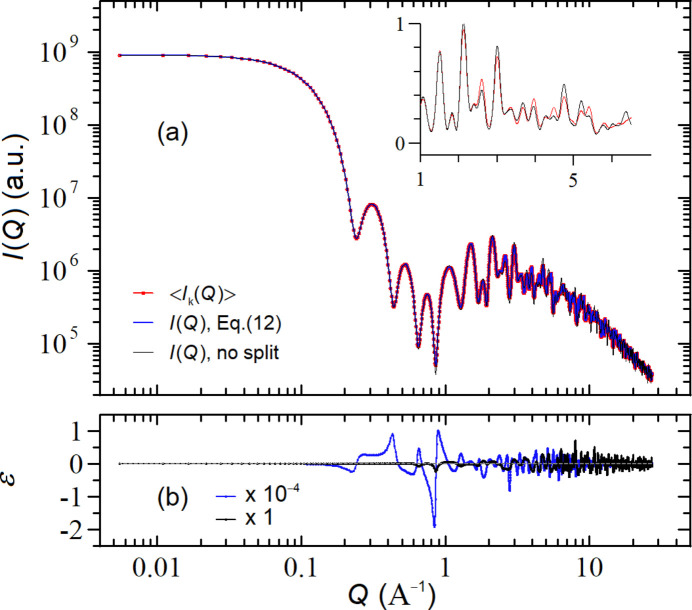
(*a*) Comparison between the expected 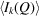 (red symbols) obtained by averaging the intensity profiles of many defective cubic CsPbBr_3_ perovskite NCs where the Br atoms were randomly displaced crosswise (see text) and the *I*(*Q*) computed via equation (12) (blue curve). For completeness, the 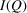 relative to an ideal CsPbBr_3_ NC (unsplit) is also reported (black curve); in the inset the same (rescaled) data are shown on a (reduced range) linear plot. (*b*) Relative deviations ɛ between 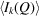 and the two *I*(*Q*) curves of panel (*a*). The factors appearing in the legend indicate the absolute scales of the *y*-axis values.

Fig. 4 ▸(*a*) compares 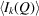 (red symbols) with the profile 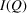 computed by using equation (12) (blue curve) where 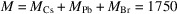 . The same (rescaled) profiles are shown in the inset of Fig. 4 ▸(*a*) on a (reduced range) linear plot. As in the previous example, the almost perfect match of the two curves [indistinguishable in Fig. 4 ▸(*a*) and the corresponding inset], with a small (dis)agreement parameter 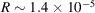 and non-systematic deviations [shown in Fig. 4 ▸(*b*)], demonstrates the correctness of equation (12). In order to highlight the effects of the splitting in the Br positions, we also compare 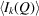 with the 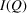 relative to an ideal CsPbBr_3_ NC, where the Br atom (small black circle in Fig. 1 ▸) is located midway along the two Pb atoms (black curve). Although the differences between 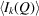 and the 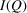 ’s relative to the two models are barely visible in Fig. 4 ▸(*a*) and in the corresponding inset, the match to 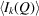 is much worse for the unsplit case [as shown in Fig. 4 ▸(*b*)], with 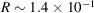 . Worthy of note, it is exactly the difference in peak intensities [rather than in positions, see inset of Fig. 4 ▸(*a*)] appreciable only with high-quality (*e.g.* synchrotron) X-ray data that paved the way to the interpretation of the data in favor of the split-cubic model at the expense of the 100% ordered one (Hanusch *et al.*, 2014 ▸; Protesescu *et al.*, 2016 ▸, 2017 ▸; Lignos *et al.*, 2018 ▸).

## 5. Discussion

In this section we address two important issues related to: (i) types and extent of errors made in recovering the NP parameters if data analysis of defective NPs is carried out by using the original MDSE (Cervellino *et al.*, 2010 ▸) instead of the corrected one [equation (7)]; (ii) comparison of the 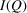 profiles associated to NPs with constant site-occupancy probability versus constant number of atoms.

### 5.1. Error estimation when using the original MDSE instead of the corrected one

**Figure 5:**
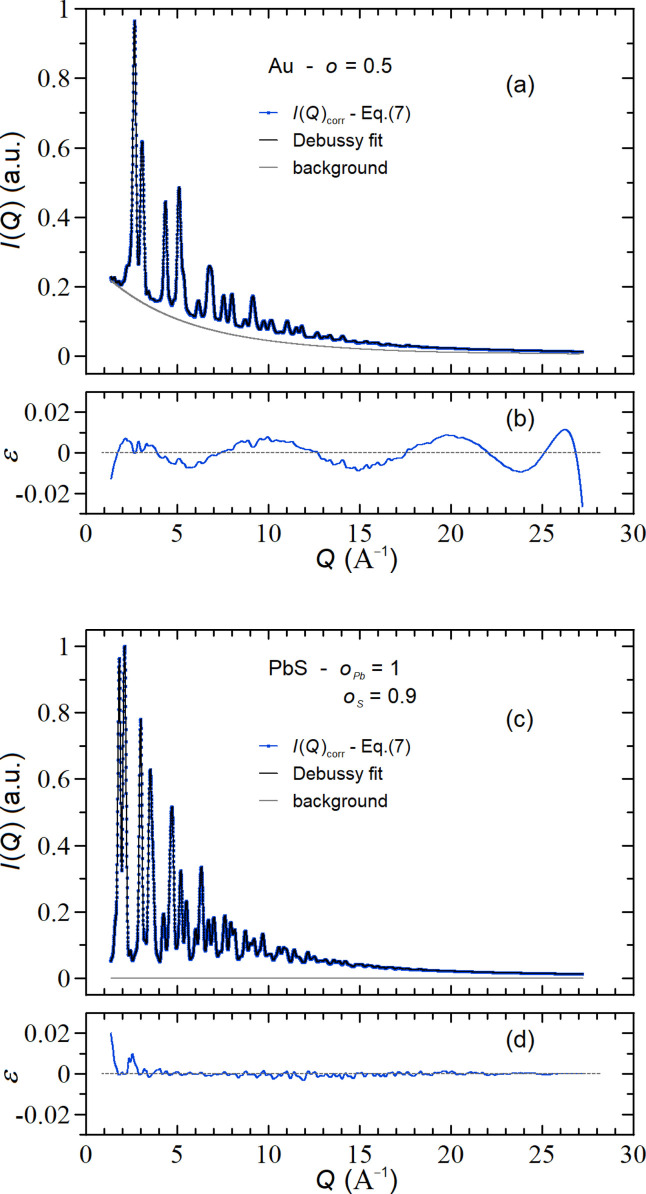
Comparison between the *I*(*Q*)_corr_ profiles computed with the corrected MDSE [equation (7), blue curves] and the background-adjusted original MDSE (black curves) for defective Au (*a*) and PbS (*c*) NCs (see Sections 3.1 and 3.2). Panels (*b*) and (*d*) show the relative residuals between *I*(*Q*)_corr_ profiles and the original MDSE-based fits. In the case of PbS, a zero background level was imposed [gray line of panel (*c*)].

To evaluate and quantify the errors from previous data analyses performed by using the original MDSE instead of the corrected one, we focus on the first two examples of the previous section. To this purpose, we analyzed the 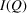 profiles generated by using the corrected MDSE [the blue curves of Figs. 2 ▸(*a*) and 3 ▸(*a*), almost identical to the simulated data] and compared them with those computed by the original MDSE. This analysis was carried out over a *Q* range typical of wide-angle X-ray total scattering (WAXTS) data (1.4–27.2 Å^−1^) by using the ‘standard’ *DEBUSSY* suite (Cervellino *et al.*, 2015 ▸). The results are summarized in Figs. 5 ▸(*a*)–5(*b*) and Figs. 5 ▸(*c*)–5(*d*) for the two examples described in Sections 4.1 and 4.2, respectively.

Fig. 5 ▸(*a*) shows that, in the case of the Au NC, the profile 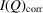 (blue curve) can be accurately reconstructed by using as fitting function (black curve) the profile given by the original MDSE plus a background profile (gray curve) optimized by using the Chebyshev polynomials with seven coefficients. The result is quite satisfactory as showcased in Fig. 5 ▸(*b*) where the relative residuals 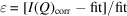 are well balanced around zero and the overall (dis)agreement parameter is 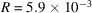 . Consistently with this result, the recovered NC crystallographic and size parameters match quite well the input ones, namely 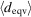 = 3.04 nm, 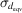 = 0 nm and *B* = 0.5 Å^2^.

Similarly, for the PbS NC, Fig. 5 ▸(*c*) shows that the profile 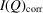 (blue curve) can be accurately reconstructed (black curve) by using the original MDSE profile only. Indeed, in this (much more realistic) case, the original and corrected MDSE are so close [see Fig. 3 ▸(*a*)] that no background contribution (gray curve) has been added, as shown by the residual plot of Fig. 5 ▸(*d*), with 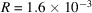 . NC crystallographic and size parameters are fully recovered also in this case, *i.e.* 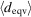 = 3.67 nm, 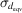 = 0 nm, 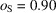 , *B*_Pb_ = 0.5 Å^2^ and *B*_S_ = 0.5 Å^2^.

Finally, we would like to comment on the fact that the use of a polynomial background is sufficient for compensating the difference between the 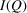 ’s computed via the corrected (*o*) and the original ( 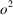 ) MDSE. This is not just a mere coincidence, but it works because this difference is a curve with a very smooth *Q* dependence that is proportional to the average NP atomic form factors, which are known to be accurately approximated by high-order polynomial functions (Freeman & Smith, 1958 ▸).

### 5.2. Constant site-occupancy probability versus constant number of atoms

As already reported in Sections 3.1 and 3.2, the 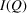 profiles associated to NPs with constant site-occupancy probability and constant number of atoms are described by equations (7) and (10), respectively. These two types of defectiveness are typically associated to NP systems characterized by quite different physical–chemical conditions: (i) the constant site-occupancy probability is linked to a thermodynamically equilibrated system, where NPs of different stoichiometries can be formed. This can occur, *e.g.*, in nano-alloys (Andreazza *et al.*, 2015 ▸; Front & Mottet, 2021 ▸) and, in the organic chemistry fields, in ideal solid solutions like the ivermectin drug (Shubin *et al.*, 2021 ▸). (ii) The fixed (randomly distributed) number of atoms is the common approach in computational chemistry, where stochastic, uncorrelated configurations are studied, and ranked in terms of energetic criteria. This is the case of binary or ternary iron oxides, in the form of maghemite (γ-Fe_2_O_3_) or tripuhyite (FeSbO_4_). In both systems, ideal periodicity is broken by the presence of vacant sites in the former (Grau-Crespo *et al.*, 2010 ▸) or partially correlated short-range ordering in the latter (Grau-Crespo *et al.*, 2004 ▸). In these solids, a constant number of metal ions (for a definite size), granting NC electroneutrality, is required.

**Figure 6:**
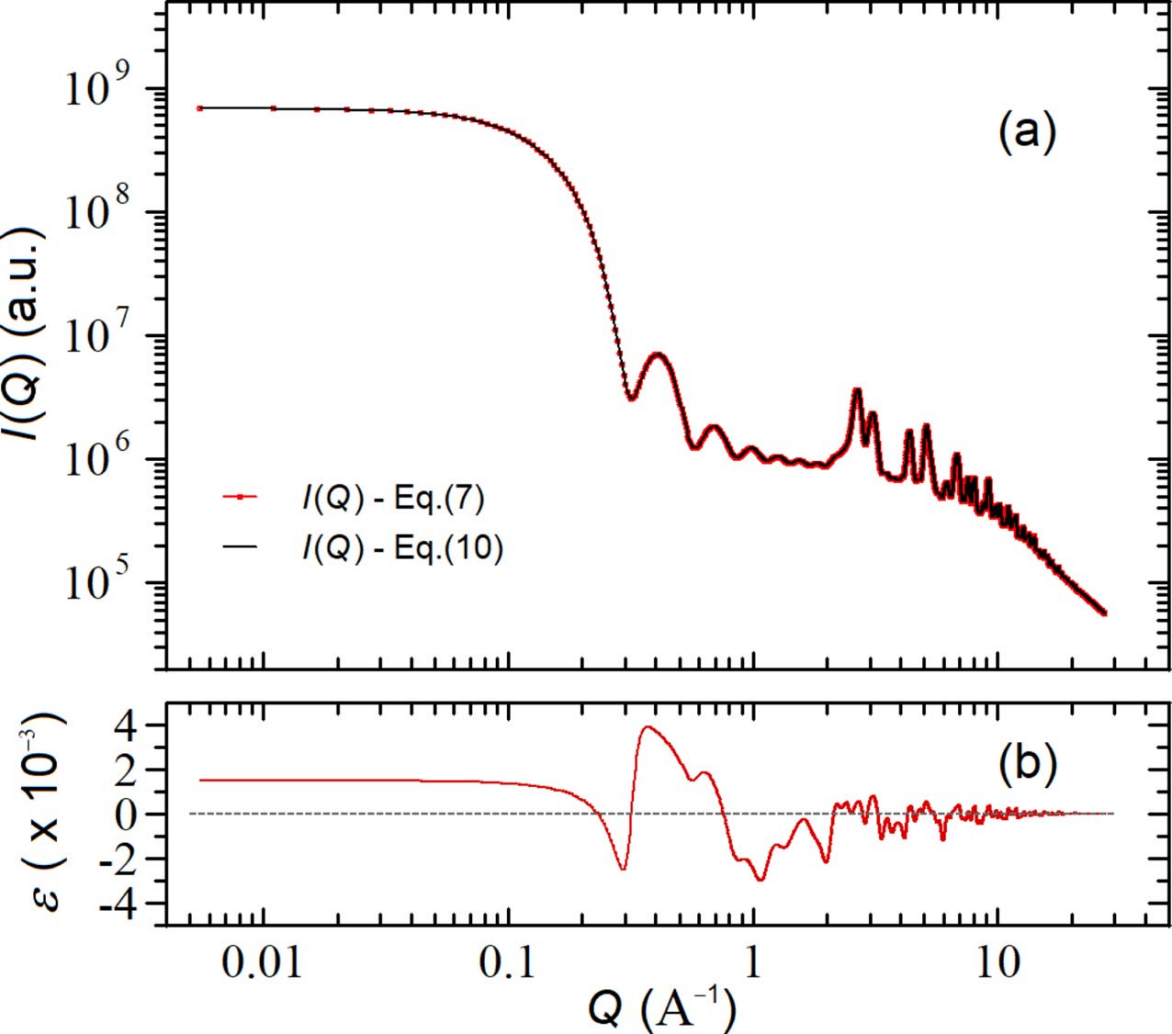
(*a*) Comparison between the 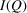 profiles of defective f.c.c. Au NPs (*a* = 4.08 Å, *M* = 666) computed with equation (7) (constant occupancy probability 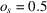 , red curve) and equation (10) (constant number of atoms 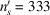 , black curve) so that 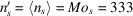 ; (*b*) relative deviations ɛ between the two curves, where ɛ = [*I*(*Q*)_Eq.(7)_ − *I*(*Q*)_Eq.(10)_]/*I*(*Q*)_Eq.(10)_.

Fig. 6 ▸ compares the theoretical profiles 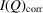 given by equations (7) and (10) relative to a f.c.c. Au NC (*a* = 4.080 Å) in the form of a cube with five unit cells per edge, characterized by *M* = 666 sites. In the case of constant occupancy probability, we set *o* = 0.5 so that 〈*n*〉 = *Mo* = 333, whereas for a (randomly distributed) constant number of atoms, we set 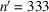 . As one can easily notice, the two curves are indistinguishable on the log–log plot of Fig. 6 ▸(*a*), but exhibit systematic deviations ( 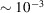 ) at small *Q*’s, as shown in the relative residual plot of Fig. 6 ▸(*b*), where ɛ = 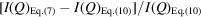 . We can further notice that, while these deviations tend to zero for 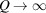 (because 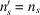 ), they remain systematically positive for 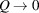 . Indeed, by studying the asymptotic behaviors of equations (7) and (10) for 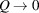 we obtain

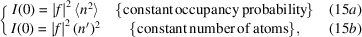

where in equation (15*a*) we have used the property that the variance of a binomial distribution is 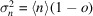 . Equations (15) show that, in the 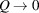 limit, the intensity scales as the square of the average total number of atoms within the NP, consistent with the fact that at zero angle the scattering amplitudes of all the atoms sum up coherently, regardless of their spatial arrangements. However, there is an important difference between the two cases: while for equation (15*b*) the number of atoms is constant, for equation (15*a*) it is a stochastic variable and therefore 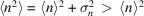 .

As a consequence, NP ensembles with constant occupancy probability exhibit a higher scattered intensity for 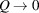 , the extra contribution being due to the fluctuations of the number of atoms inside the NPs. Such an extra contribution is given by 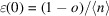 (valid for a binomial distribution) and, therefore, under most (realistic) conditions where 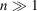 , can be neglected [ 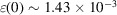 for the curve of Fig. 6 ▸(*b*)]. We conclude that, unless ultra-small NPs with sizes of ∼1–2 nm are considered, the two types of defectiveness are indistinguishable in a standard WAXTS analysis where the *Q* range typically starts from *Q*_min_ ≥ 0.5 Å^−1^.

## 6. Conclusions

In this paper we have revised (and corrected) the original modified Debye scattering equation (Cervellino *et al.*, 2010 ▸) by providing theoretical justification of using atomic s.o.f.’s as free adjustable parameters and presenting cases of application to a variety of defective NP ensembles. For all these cases, we thoroughly discussed the significance of the s.o.f.’s 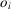 ’s, provided the analytical expression for the 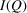 profiles, and showed that the 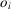 ’s appearing in the 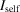 contribution of the original MDSE must not be squared. The revised versions of MDSE are suitable for describing:

(i) Defective NP ensembles with vacant sites with *uncorrelated constant site-occupancy probability* which describes systems at thermodynamic equilibrium where the number of atoms is not constant, but varies stochastically according to the binomial distribution described in Section 3. The expression for the 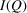 profile is given by equation (7).

(ii) Defective NP ensembles with a *constant number of randomly distributed atoms* that are imposed by stoichiometric constraints. In these systems, the number of atoms of each species is fixed and smaller than the number of available sites for that species. The expression for the 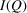 profile is given by equation (10).

(iii) Defective NP ensembles associated to a special (but common) case of correlated occupancies, *i.e.* the ones with *self-excluding disordered positional sites* where the sites belonging to a given atomic species can be split in different clusters, each of them comprising only one atom (a ‘split-atom’ model). In this case, the 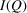 profile is computed by restricting the 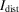 contribution to distances belonging to different clusters and is given by equation (12).

As already pointed out in Section 3.1, the main difference between the original MDSE expression and the corrected one is the presence of (wrong) squared s.o.f.’s appearing in 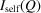 . This glitch reduces the continuous and smooth contribution of 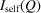 to the overall scattering profile but does not affect the 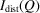 term from which the NP structural characterization is extracted. Therefore, the effect on the total intensity 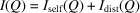 is a reduction of the diffuse scattering and a corresponding spurious enhancement of the Bragg peaks. The relative discrepancy between the original and corrected MDSE profiles may be compensated by the usage of a smoothly changing polynomial function acting as a ‘background’ contribution. Our numerical simulations performed on highly defective Au NCs show that this background adjustment is very effective and allows the accurate recovery of the NC crystallographic parameters. More importantly, under realistic conditions, that is with almost unitary occupancy factors in non-elemental systems (less defective PbS NCs), such an additional background adjustment is not required at all and these (negligible) effects do not affect previously published results.
